# A high-throughput method to detect *Plasmodium falciparum *clones in limiting dilution microplates

**DOI:** 10.1186/1475-2875-11-124

**Published:** 2012-04-24

**Authors:** Brian Lyko, Elizabeth A Hammershaimb, Wang Nguitragool, Thomas E Wellems, Sanjay A Desai

**Affiliations:** 1 The Laboratory of Malaria and Vector Research, National Institute of Allergy and Infectious Diseases, National Institutes of Health, Rockville, MD 20852, USA; 2 Faculty of Tropical Medicine, Mahidol University, Bangkok, Thailand; 3 Laboratory of Malaria and Vector Research, NIAID/NIH, Room 3W-01, 12735 Twinbrook Parkway, Rockville, MD 20852-8132, USA

## Abstract

**Background:**

Molecular and cellular studies of *Plasmodium falciparum *require cloning of parasites by limiting dilution cultivation, typically performed in microplates. The parasite's slow replication rate combined with laborious methods for identification of positive wells has limited these studies. A new high-throughput method for detecting growth without compromising parasite viability is reported.

**Methods:**

*In vitro *parasite cultivation is associated with extracellular acidification. A survey of fluorescent pH indicators identified 5-(and-6)-carboxy SNARF-1 as a membrane-impermeant dye with a suitable *pK_a _*value. Conditions for facile detection of viable parasites in 96-well microplates were optimized and used for limiting dilution cloning of genetic cross progeny and transfected parasites.

**Results:**

5-(and-6)-carboxy SNARF-1 is a two-emission wavelength dye that accurately reported extracellular pH in parasite cultures. It readily detected parasite growth in microplate wells and yielded results comparable to labour-intensive examination of Giemsa-stained smears. The dye is non-toxic, allowing parasite detection without transfer of culture material to additional plates for separate assays. This dye was used with high-throughput limiting dilution culture to generate additional progeny clones from the HB3 × Dd2 genetic cross.

**Conclusions:**

This fluorescence-based assay represents a low-cost, efficient method for detection of viable parasites in microplate wells; it can be easily expanded by automation.

## Background

Molecular and genetic studies have provided fundamental insights into malaria parasite biology; with continuing advances in underlying technologies, progress in these studies will likely accelerate. Because *Plasmodium falciparum *cultivation is performed in suspension cultures, these studies require efficient methods to obtain clonal lines.

Limiting dilution is frequently employed for cloning, typically in 96-well microplates with less than one parasite/well in the presence of uninfected erythrocytes [1]. After cultivation for two to three weeks, parasite growth in individual microplate wells is detected by one of several methods, each of which has one or more shortcomings. The method used by most laboratories, examination of smears stained with Giemsa or modified Field's stain, is labour-intensive, requires training to confidently identify parasites, and cannot be scaled up beyond a few microplates. Other commonly used methods are based on detection of parasite DNA, a secreted antigen, or specific enzymatic activity [2-4]. These methods are sensitive, but each requires partial transfer of the culture to assay plates and subsequent procedures for detection. These steps of transfer and experimental detection add to the total assay cost, can lead to erroneous calling of positive wells, raise risks of contamination, and consume precious small-volume cultures. Another method, visual examination of parasite growth in microplate wells [5], has not achieved broad use because the subtle colour changes can be difficult for investigators to confidently recognize.

Here, a novel fluorescence-based method for detection of viable parasites in microplates is reported. The method is simple to perform with standard fluorescence plate readers, does not require microplate transfer, and can be readily scaled for high-throughput studies.

## Methods

### Parasite culture and detection of parasites with 5-(and-6)-carboxy SNARF-1

*Plasmodium falciparum *was cultivated by standard methods. Cryopreserved chimpanzee blood from a previous HB3 × Dd2 genetic cross was cultivated in human O^+ ^erythrocytes for two to 10 days prior to initiation of cloning by limiting dilution.

Limiting dilutions were set up in 96-well plates (Costar flat bottom cell culture plates, Corning, Lowell, MA, USA) at 2% haematocrit in RPMI 1640 supplemented with 10% v/v pooled human serum, 28.6 mM NaHCO_3_, 10 μg/mL gentamicin, and 25 mM HEPES, pH 7.4. Each well contained 200 uL of medium and an average of 0.1-0.5 parasites. Medium changes (175 uL) were performed at two- or three-day intervals beginning with day 5. On or after day 8, changes used HEPES-free medium that contained 1 μM 5-(and-6)-carboxy SNARF-1 (c-SNARF-1, Invitrogen, Carlsbad, CA, USA). Use of HEPES-free medium improved parasite detection by reducing the extracellular buffering capacity; it did not noticeably compromise parasite viability. Positive wells were also readily detected in experiments that used HEPES-containing medium. Parasite growth was assessed by c-SNARF-1 fluorescence (excitation 485 nm, emission 590 and 645 nm; Synergy HT plate reader, BioTek, Winooski, VT, USA) before each medium change. Precautions to avoid photobleaching of c-SNARF-1 by ambient light were unnecessary. Microplates were equilibrated to room temperature (≤ 20 min) prior to fluorescence measurements to minimize effects of temperature- and CO_2_-gradients across the plate. Wells with a significant increase in the 590/645 nm emission ratio were transferred to standard culture flasks for expansion of clonal cultures; depending on parasite growth rate, positives could be detected as early as 13 days after initiating limiting dilution. Addition of 5 nM WR99210 or 2.5 μg/mL blasticidin S, as often used with selectable markers in parasite DNA transfections, did not adversely affect identification of parasite clones with c-SNARF-1. Because c-SNARF-1 fluorescence intensity is stable under culture conditions (~5% decrease over 7 days), the assay is compatible with less frequent media changes than used here. 1% w/v Albumax II, used as a substitute for human serum in parasite cultures, quenched c-SNARF-1 fluorescence by approximately 50%, but did not adversely affect ratiometric pH measurements.

Calculation of the 590/645 nm fluorescence ratio, generation of a graphical output, and identification of positive wells were automated using scripts written in SigmaPlot 10.0 (Systat, San Jose, CA); these scripts are available upon request.

### SYBR Green I assays

Parasite growth inhibition studies were performed using a SYBR Green I-based fluorescence assay for parasite nucleic acid in 96-well format, as described previously [6]. Sorbitol synchronized cultures were prepared at 1% parasitaemia and 2% haematocrit in media without or with c-SNARF-1 as indicated. When required, the pH of the medium was adjusted with HCl or NaOH to indicated values after addition of HEPES and NaHCO_3_. After maintenance at 37°C in 5% O_2 _and 5% CO_2 _for indicated durations, the cultures were lysed in 20 mM Tris, 10 mM EDTA, 0.016% saponin, and 1.6% triton X100, pH 7.5 with SYBR Green I nucleic acid gel stain at a 5,000x dilution (Invitrogen, Carlsbad, CA, USA). After a 45-min incubation, parasite DNA content was quantified by measuring fluorescence (excitation/emission wavelengths, 485/528 nm). Mean parasite growth was calculated from triplicate measurements after subtraction of background fluorescence from parallel cultures killed by incubation with 20 μM chloroquine. Control experiments revealed that SYBR Green I fluorescence is pH-independent and not affected by cross-talk with c-SNARF-1.

## Results and discussion

### c-SNARF-1 has excellent fluorescence output and an optimal *pK_a _*for detection of parasite growth

**Figure 1:**
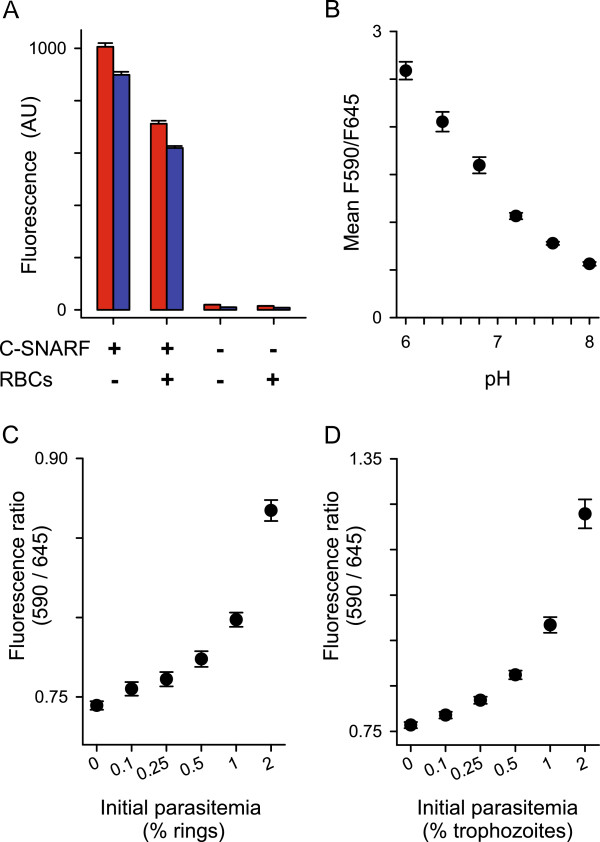
**Detection of parasite metabolic acid production with c-SNARF-1**. **(A) **Fluorescence measurements in microplate wells containing RPMI 1640 with phenol red and 10% serum; 5 μM c-SNARF-1 or erythrocytes (5% haematocrit) were added as indicated. Red and blue bars represent mean ± S.E.M. fluorescence emission at 590 and 645 nm, respectively. Notice the marked increase in fluorescence at each emission wavelength when the dye is present. AU, arbitrary units. **(B) **The ratio of fluorescence output as a function of pH in complete medium. Identical results were obtained with 1, 5, and 20 μM c-SNARF-1 (not shown). **(C-D) **Mean ± S.E.M. fluorescence ratios in microplate wells seeded with indicated initial parasitaemia and cultivated for 48 h. Note the greater sensitivity for detection of trophozoite-stage parasites. 1 μM c-SNARF-1, 2% haematocrit.

A search for methods to detect parasite metabolic acid identified c-SNARF-1 as a long-wavelength fluorescent pH indicator with a *pK_a _*of 7.5 [7]. This value suggested that it could sensitively detect changes in extracellular pH associated with parasite growth. This dye was also attractive because it undergoes a pH-dependent shift in emission wavelength, permitting ratiometric measurements that are independent of variations in dye concentration. Figure 1A shows measured fluorescence intensities at 590 and 645 nm, values near the reported emission maxima under acidic and basic conditions, respectively. There was negligible fluorescence at either wavelength from erythrocytes or culture medium containing serum and phenol red. c-SNARF-1, added at a 5 μM concentration, produces a marked increase in fluorescence output at both wavelengths. This output is partially quenched by addition of erythrocytes, but the signal-to-noise ratio permits routine use of the dye at a 1 μM concentration. The ratio of fluorescence output at these wavelengths responded sensitively to pH in culture media (Figure 1B). These findings indicate a low background and suggested the dye is well-suited for detection of changes in extracellular pH.

To determine whether 1 μM c-SNARF-1 can serve an as indicator of parasite metabolism, 48-h cultivation experiments were initiated with synchronous ring- or trophozoite-stage *P. falciparum *cultures. These studies revealed significant increases in the fluorescence ratio relative to control wells containing only uninfected cells, indicating reliable parasite detection (Figure 1C-D, *P *< 0.01 for 0.25% parasitaemia for either ring- or trophozoite-stage cultures). The ratio increased monotonically with parasitaemia, consistent with parasite-specific production of lactic acid and acidic metabolites. Interestingly, trophozoite-infected cells produced greater changes despite use of a 48-h incubation that allows for a complete intra-erythrocytic cycle, possibly reflecting stage-specific production of these acids [8].

### c-SNARF-1 and decreases in extracellular pH are non-toxic to parasite cultures

**Figure 2:**
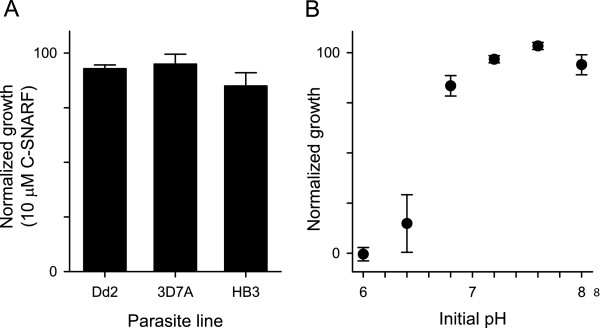
**The assay is non-toxic to parasite cultures**. **(A) **Growth of indicated parasite lines over five days in the presence of 10 μM c-SNARF-1, quantified with SYBR Green I and normalized to 100% growth for controls grown without c-SNARF-1. Bars represent mean ± S.E.M of three to four trials each. **(B) **Normalized parasite growth in HEPES- and NaHCO_3_-buffered media with indicated initial pH values (*n *= 3). Cultures were initiated at 1% synchronous ring-stage parasitaemia and assessed with SYBR Green I after 72 h.

Parasite cultivation with a fluorescent dye obviates microplate transfers; however, the dye must be non-toxic to parasite cultures. Using a high 10 μM concentration in five-day growth experiments, c-SNARF-1 had negligible effects on expansion of three common parasite lines with distinct anti-malarial susceptibilities (Figure 2A); microscopic examination of smears also revealed unaltered parasite morphology after cultivation with this dye. These parasites are from three different continents, suggesting that diverse *P. falciparum *clones will not be adversely affected by the lower 1 μM c-SNARF-1 concentration used for detection of parasite growth and limiting dilution cloning.

To determine if the drop in extracellular pH required to produce a significant change in c-SNARF-1 output is detrimental to parasite growth, parasite propagation was examined in media having a range of pH values (Figure 2B). These studies showed growth over a broad pH optimum, with cultures initiated at a pH of 6.8 growing nearly as well as those in standard media with a pH of ~7.3. Changes in pH over this range were readily identified by c-SNARF-1 (Figure 1C-D), indicating that this dye can report parasite growth without compromising viability.

### Detection of parasites clones from limiting dilutions in microwell plates

**Figure 3:**
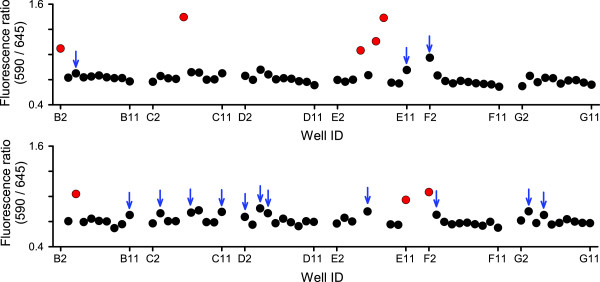
**Identification of limiting dilution parasite clones**. Fluorescence ratio determinations from a single 96-well microplate, read on days 19 and 21 of limiting dilution culture (top and bottom panels, respectively). Red symbols reflect positive wells that were harvested. Note that the weak positives in the top panel (blue arrows) exhibited greater increases after continued culture (red symbols in the bottom panel); additional weak positives in the bottom panel were also confirmed on subsequent reading (not shown). These positives likely represent medium- to slow-growing clones from the HB3 × Dd2 cross. Negative wells exhibit low variability. Edge wells were not used in this experiment.

Limiting dilution cloning was next performed in the presence of 1 μM c-SNARF-1, yielding unambiguous identification of parasite growth in 96-well microplates (red symbols, Figure 3); these parasites were successfully expanded after transfer to culture flasks. Wells that had modestly elevated fluorescence ratios produced greater increases after continued culture in the microplate (blue arrows), consistent with a correlation between expansion of cultures and greater decreases in extracellular pH. To obtain maximal recovery of clones from microplates, parasite growth was assessed prior to each medium change, which is routinely performed at two-day intervals. Slow-growing parasite clones were detected as late as 39 days in the continued presence of c-SNARF-1, excluding accumulated toxicity from dye exposure.

Sensitivity and specificity of the c-SNARF-1 assay were compared to microscopy of Giemsa-stained smears with limiting dilution experiments using eight microplates. From these plates, 39/480 wells were positive with c-SNARF-1; all of these wells were positive by microscopy. Two additional wells were implicated by microscopy, but these were later judged to be false positives as subsequent smears failed to detect parasites. While these methods have comparable sensitivity, c-SNARF-1 typically identified positive wells several days later than microscopic examination of smears. In practice, delayed identification is not a disadvantage: most molecular or phenotypic studies require a large number of parasites, as obtained by expansion of clones in culture flasks. Because c-SNARF-1 does not affect parasite expansion (Figure 2A), the time to reaching that number does not depend on when limiting dilution clones are detected.

To determine if sensitive detection of clones can be carried out in a high-throughput format, limiting dilution was used to generate additional progeny clones from the HB3 × Dd2 *P. falciparum *genetic cross [9]. Two experiments using a total of 36 microplates yielded 203 positive wells. Genotyping of these parasites is pending, but a significant increase in the number of independent recombinant progeny clones from this cross is anticipated. These progeny clones should be useful for examination of parasite phenotypes having complex inheritance patterns and are available upon request. Notably, a single investigator carried out both the limiting dilution cloning and the c-SNARF-1 detection of positive wells. The negligible effort required to identify parasite growth with this assay should support a variety of high-throughput studies.

## Conclusions

The new c-SNARF-1 assay represents an improvement over available methods of detecting parasite growth in microplates. Because this dye is non-toxic, the assay does not require microplate transfer, decreases risks of contamination, and reduces both the required labour and costs associated with disposable supplies. The assay can be performed with minimal effort prior to each regularly scheduled medium change, ensuring sensitive and timely harvest of clones. High-throughput detection of progeny clones from an existing genetic cross demonstrates the potential of this assay to contribute to malaria research.

## Competing interests

The authors declare that they have no competing interests.

## Authors' contributions

BL, EAH, and WN designed and performed experiments and carried out data analysis. TEW provided reagents and guidance on limiting dilution. SAD conceived the project, analysed data, and wrote the paper. All authors read, revised and approved the manuscript.

## Funding

This research was supported by the Intramural Research Program of the National Institutes of Health, National Institute of Allergy and Infectious Diseases.
